# The Brain Functional State of Music Creation: an fMRI Study of Composers

**DOI:** 10.1038/srep12277

**Published:** 2015-07-23

**Authors:** Jing Lu, Hua Yang, Xingxing Zhang, Hui He, Cheng Luo, Dezhong Yao

**Affiliations:** 1 Key Laboratory for NeuroInformation of Ministry of Education, School of Life Science and Technology, University of Electronic Science and Technology of China; 2 Department of Composition, Sichuan Conservatory of Music.

## Abstract

In this study, we used functional magnetic resonance imaging (fMRI) to explore the functional networks in professional composers during the creation of music. We compared the composing state and resting state imagery of 17 composers and found that the functional connectivity of primary networks in the bilateral occipital lobe and bilateral postcentral cortex decreased during the composing period. However, significantly stronger functional connectivity appeared between the anterior cingulate cortex (ACC), the right angular gyrus and the bilateral superior frontal gyrus during composition. These findings indicate that a specific brain state of musical creation is formed when professional composers are composing, in which the integration of the primary visual and motor areas is not necessary. Instead, the neurons of these areas are recruited to enhance the functional connectivity between the ACC and the default mode network (DMN) to plan the integration of musical notes with emotion.

As an important feature of human beings, creativity has long held the attention of scientists and psychologists. Although studies of creativity have a long history in psychology^1^,^2^, research on the topic remains problematic. Until now, it has been universally accepted that creative products share two characteristics: they are novel and they are meaningful. Several cognitive processes are engaged during creative thinking, including attention to action, response generation^3^, action planning and monitoring, and inhibition of repetitive responses^4^. Although the specific neural mechanisms related to creativity remain unclear, different brain regions appear to interact in these processes, indicating that creativity relies on distributed networks^5^.

Music is an art shared by all human beings^6^. Emotional expression and communication through music are recognized to be strongly linked to health and a sense of well-being. Music appreciation is the generative capacity that allows individuals to enjoy music, whether the piece is melodious or passionate^7^. For several decades, scientists and musicologists have devoted themselves to discovering the relationship between music and the human experience^8^. In recent years, music has been considered to be an outstanding tool for improving fundamental understanding of the human brain, and many studies have investigated the relationship between music and the human brain^9^,^10^,^11^,^12^.

Music creation, as an artistic endeavor, is usually considered one of the most mysterious forms of creativity. It is often described as occurring in an altered state of mind beyond conscious awareness in the human brain^13^,^14^,^15^. Until now, several studies have focused on the neural effects of creating music as a model to discover the relationship between creativity and the human brain. In 2007, Bengtsson *et al.* used fMRI to study brain activity during music creation. They asked 11 pianists to improvise a melody and then reproduce it on a small keyboard. During improvisation, activated brain regions were found in the right dorsolateral prefrontal cortex, the presupplementary motor area, the rostral portion of the dorsal premotor cortex, and the left posterior part of the superior temporal gyrus compared to the reproduction period^16^. In 2008, Limb and Braun recruited 6 jazz pianists to improvise on a piano keyboard during fMRI scan. They used two paradigms that differed widely in musical complexity, and found that improvisation was consistently characterized by a dissociated pattern of activity in the prefrontal cortex^17^. As in Limb’s experiment, in 2014 Donnay *et al.* invited jazz pianists to improvise during fMRI scan. They examined an interactive improvisation between two musicians, and the results showed that the activation of perisylvian language areas was linked to the processing of syntactic elements in music^18^. In the same year, Peter *et al.* discussed auditory feedback during musical production on a keyboard and found evidence of neural responses associated with perception/action mismatch during improvisation by fMRI^19^.

From these studies, we can conclude that the neural mechanisms of music creation are not systematic, and even scientists consider the current results to be controversial. In addition, music creation is a complicated process^20^,^21^, and the focus on pianists in previous studies is limiting. As we know, performance and composition are two different stages in the creation of music. Pianists are performers, who usually create music through experiences and memories. Therefore, their improvisation cannot represent a purely compositional form of music creation.

In this study, we recruited composition majors and subjected them to common fMRI methods. Moreover, because most of the composers could play piano, we asked subjects to compose a piece of music for an instrument that they do not play (‘Chinese Zheng’) during the task period. Thus, we could eliminate the effect of motor experience and memory. We aimed to identify the brain functional state in composers from the network perspective, and to further our understanding of how creative behaviors are processed in the brain.

## Methods

### Ethics statement

All participants gave their written, informed consent in compliance with an experimental protocol approved by the Ethics Committee of the School of Life Science and Technology at the University of Electronic Science and Technology of China (UESTC). Experiments were carried out in accordance with the approved guidelines. All the subjects participating in the experiment gave informed consent before the experiment was conducted according to the established guidelines of the Ethics Committee of School of Life Science and Technology at UESTC.

### Subjects

Seventeen composers (12 female, *M*_*age*_ = 27.18 ± 5.01 years, who have composed for more than 5 years for over 4 hours a day) from the Sichuan Conservatory of Music participated in this experiment. They were all right-handed according to the Edinburgh Inventory^22^ with normal hearing, normal vision, and no history of neurological disorders. All composers reported the instruments they could play (none of them could play the ‘Chinese Zheng’).

### Image data acquisition

Images were acquired on a 3T magnetic resonance imaging (MRI) scanner (GE Discovery MR750, USA) at the MRI Research Center of UESTC using a standard GE whole head coil.

First, we conducted a resting state fMRI scan on composers. During scanning, we used foam padding and ear plugs to reduce head motion and scanning noise, respectively. Resting state functional MRI data were acquired using gradient-echo EPI sequences (repetition time [TR] = 2000 *msec*, echo time [TE] = 30 *msec*, flip angle [FA] = 90°, matrix = 64 × 64, field of view [FOV] = 24 × 24 *cm*^2^, slice thickness/gap = 4 *mm*/0.4 *mm*), with an eight channel-phased array head coil. All subjects underwent a 510 second resting state scan to yield 255 volumes (35 slices per volume). During resting state fMRI, all subjects were instructed to close their eyes and to move as little as possible without falling asleep. Subsequently, high-resolution T1-weighted images were acquired using a 3-dimensional fast spoiled gradient echo (T1-3D FSPGR) sequence (TR = 5.948 *msec*, TE = 1.964 *msec*, FA = 9°, matrix = 256 × 256, FOV = 20.4 × 16.3 cm^2^, slice thickness/gap = 1 mm/0 mm, 154 slices).

**Figure 1:**
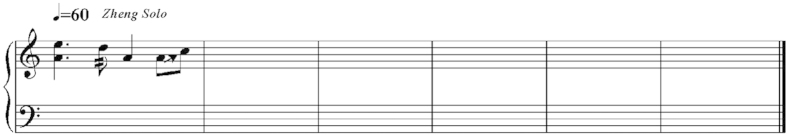
The stave that was used in the experiment.

After the resting state fMRI scan, we conducted another scan, which used the same imaging parameter as above and lasted for 5 minutes. Before the scan, subjects were given instructions on how to perform the experiment. In this scan, composers could see one page of stave. To explore the creation of music, they were told to compose a piece of ‘Chinese Zheng’ music with beginning tones using their imagination (imaging composition; material is shown in Fig. 1). The beginning tones were composed by Hua Yang, who is an established composer, so that we could ensure that none of the subjects had seen the material before. During the scan, subjects could not move in the MRI except to look at the screen and imaging composing. When the subjects exited the scan machine, they were asked to write down the imagined music on a stave paper immediately so that we could ensure the composing process had been scanned.

### Data processing

#### Pre-processing

The preprocessing and statistical analysis of fMRI data were executed with the program SPM8 (Statistical Parametric Mapping, http://www.fil.ion.ucl/spm/software/spm8). To avoid the MRI machine field effect and to eliminate head movements of the participants, we discarded the first five volumes. This procedure includes slice timing correction, head realignment and normalizing images with a EPI template from the Montreal Neurological Institute (MNI) atlas space^23^ and resampling to 3 × 3 × 3 *mm*^*3*^. Band-pass temporal filtering (0.01–0.08 *Hz*) was used to remove magnetic field drifts of the scanner and to minimize the physiologic noise of high frequency components. These images were then smoothed with an 8 mm × 8 mm × 8 mm full-width at half-maximum (FWHM) Gaussian kernel. Nuisance signal regression (White Matter [WM], Cerebro-Spinal Fluid [CSF], Global Signal [GS]) was not included for the following functional connectivity density (FCD) analysis but was included for the functional connectivity (FC) analysis.

#### FCD analysis

Tomasi proposed an alternative voxel-wise data-driven method, which is termed FCD mapping^24^,^25^. This method overcomes the limitation of seed-based approaches for identifying and locating functional hubs in the brain and is widely used to study training-related functional changes in the brains of experts, such as musicians^26^,^27^,^28^, chess players^29^ and athletes^30^. Therefore, we adopted the method to study the brains of composers, who also possess highly trained skills. In FCD analysis, the correlation threshold (*Tc*) was used to determine the significance of correlations between voxels. However, there is no standard from previous studies. Considering that *Tc* < 0.4 leads to an increased false positive rate^24^ and increased CPU time to compute the maps, we defined the *Tc* value beginning at the common choice 0.6 and reaching 0.85 in steps of 0.05. These threshold parameters were used to compute the number of functional connections between a given voxel and other voxels through correlation. Functional connections with a correlation coefficient *R* > *Tc* were considered significant. First, we used an algorithm to acquire the short-range FCD. We considered that a voxel *x*_*i*_ was a neighbor voxel of a given voxel *x*_*0*_ when *x*_*i*_ was adjacent to a voxel that was linked to *x*_*0*_ by a continuous path of functionally connected voxels and the correlation coefficient between *x*_*0*_ and *x*_*i*_ was significant. Though circular computations for all voxels adjacent to the neighbor voxels of *x*_*0*_’s cluster center, we obtained all new neighbors. Furthermore, we calculated the global FCD, which was defined as the number of significant connections between this voxel and all other voxels in the brain. Then, we calculated the difference between global FCD and the short-range FCD, which is also called the long-range FCD. It determines the number of significant connections (*R* *>* *Tc*) without local cluster restrictions. Finally, each participator’s short-range FCD maps and long-range FCD maps were smoothed with 8 mm FWHM. Voxel-wise paired *t*-tests implanted in SPM8 were used to evaluate group differences of short and long-range FCD maps between different conditions.

#### FC analysis among ROIs

To better analyze the differences between the two states, we focused on the clusters that were significantly different between the two states after four consecutive FCD comparisons of *Tc* values. Then, the intersections of significantly different clusters were used as regions of interest (ROIs). Then, we calculated the functional connectivity between the ROIs and all voxels in the whole brain. We used Fisher’s *z*-transformation to transform the resulting correlation coefficients to approximate a Gaussian distribution. We then used paired *t*-tests to define differences in functional connectivity between two different states.

## Results

### FCD results

**Figure 2:**
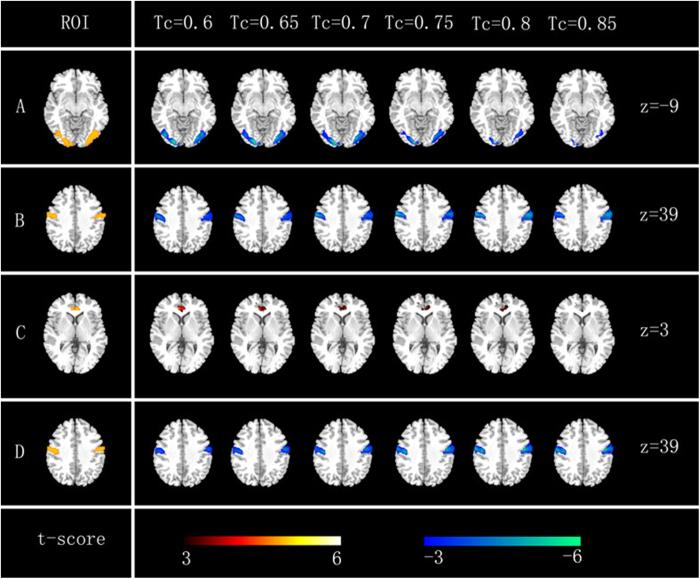
FCD result of the resting state and the composing state (from *Tc *= 0.6 to 0.85, stepped by 0.05, *p* < 0.001, cluster threshold k > 600 mm^3^). Rows ‘**A**’ and ‘**B**’: short-range FCD. Rows ‘**C**’ and ‘**D**’: long-range FCD. The five ROIs are shown in the left column. Reference color bar is shown on the bottom row.

During the FCD analysis, nobody was excluded by our standards, including head motion, behavioral indicators and psychological assessments. Thus, the resting state and the composing state were included in the final analysis. We calculated each individual’s short- and long-range FCD maps at different *Tc* values in two groups (the resting state and the composing state). In total, five ROIs were identified from the all results (Fig. 2), and their centers are listed in Table 1.

### FC results

**Figure 3:**
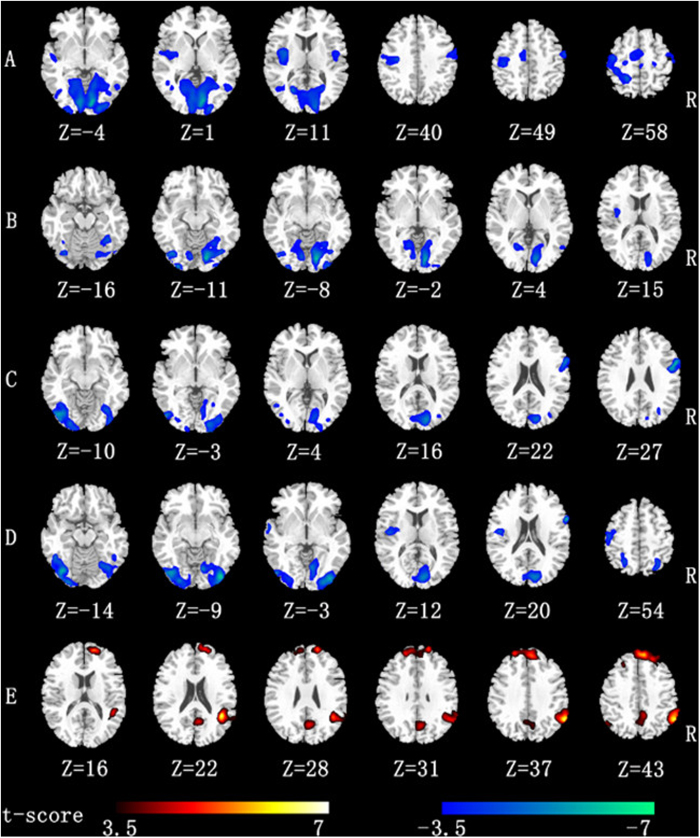
Results of functional connectivity analysis (*p* < 0.05, FDR-corrected, cluster threshold k > 600 mm^3^). Row ‘**A**’ represents the significantly decreased functional connectivity of the left occipital seed. Row ‘**B**’ reveals that of the right occipital seed. Row ‘**C**’ represents the significantly decreased functional connectivity of the left postcentral seed. Row ‘**D**’ reveals the significantly decreased functional connectivity of the right postcentral seed. Row ‘**E**’ reveals the significantly increased functional connectivity of the left cingulum seed.

We assessed the region-wise functional connectivity among five ROIs between the resting state and the composing state in composers (Fig. 3).

#### Left occipital seed

The paired *t*-test comparing the functional connectivity maps generated from the left occipital seed showed that this region had less functional connectivity in the task state than the bilateral occipital gyrus, bilateral postcentral gyrus, bilateral lingual gyrus, bilateral calcarine gyrus, bilateral fusiform gyrus and bilateral precentral gyrus.

#### Right occipital seed

In the composing state, the right occipital gyrus was negatively correlated with the bilateral lingual gyrus, right calcarine gyrus, right fusiform gyrus, right inferior occipital gyrus and left lingual gyrus.

#### Left postcentral seed

Compared with the resting state, the left postcentral gyrus in composing state was significantly decreased and was correlated with the bilateral inferior occipital gyrus, left fusiform gyrus, right calcarine gyrus, right precentral and right postcentral.

#### Right postcentral seed

Compared to the resting state, the composing state showed significantly decreased functional connections between the right postcentral gyrus and bilateral inferior/middle occipital gyrus, bilateral fusiform gyrus, bilateral calcarine gyrus, bilateral cuneus gyrus, right precentral gyrus and left postcentral gyrus.

#### Left cingulum seed

The paired *t*-test that we used to compare the functional connectivity maps for the left cingulum seed revealed a significantly stronger correlation in the composing state than in the resting state between the composers’ time series activity for this area and those of voxels located in the right precuneus, the right angular, bilateral superior medial frontal gyrus, the bilateral middle frontal gyrus and bilateral superior frontal gyrus.

From functional connectivity analysis, we found that the functional connectivity between the bilateral occipital lobe and bilateral postcentral cortex, which represent the visual area and motor area, decreased in the imaging composition state compared to the resting state. However, when composing, composers exhibited significantly stronger functional connectivity between the ACC, the right angular gyrus and the bilateral superior frontal gyrus. As we know, the right angular gyrus and bilateral superior frontal gyrus are included in the DMN^31^. Thus, we can conclude that the ACC and DMN have stronger functional connectivity during composition.

## Discussions

In our study, we used resting state fMRI to explore intrinsic functional connectivity in the brains of composers. After FCD and functional connectivity analyses, we observed several changes of the brain network during the composing period. Our results indicate that the functional connectivity of primary networks such as the visual and motor areas decreased compared to the resting state. However, some networks, such as the ACC and DMN, showed stronger functional connectivity during composition.

**Figure 4:**
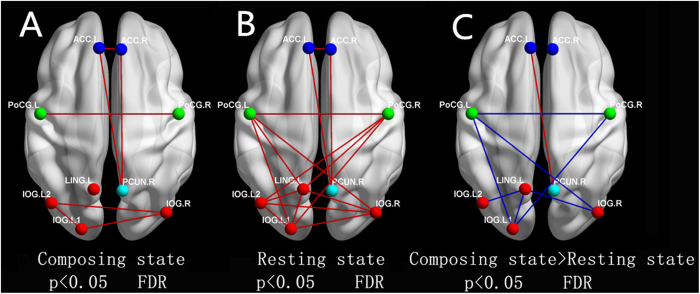
Region-wise functional connectivity between networks. ‘**A**’ and ‘**B**’ show the layout of nodes and undirected edges of networks during different states, respectively. ‘**C**’ shows the different functional connectivity between the composing state and the resting state, and we find that the functional connectivity between visual and motor areas in primary networks has decreased (blue lines), whereas the functional connectivity between the ACC and the DMN increased (red line) in the composing state.

A one-sample *t*-test was performed on all possible connections represented in the correlation matrices between the two states. For a better visualization of structural patterns of functional connectivity, nodes and undirected edges were represented as networks (Fig. 4A,B). The edges between nodes were constructed by setting a significance level of *p* > 0.05. To compare functional connectivity between each pair of nodes across the two states, two-sample two-tailed *t*-tests were performed on all potential connections included in the correlation matrix. Compared with the resting state, the functional connectivity between the visual and motor areas in primary networks decreased, whereas the functional connectivity between the ACC and DMN increased in the composing state (Fig. 4C).

### Why does functional connectivity decrease in primary networks during composition?

According to the previous study, professional pianists showed increased activity during music improvisation, including the activation of visual and motor cortex^32^. However, in our experiment, we chose to have the composers create a piece of music that was to be played on the Chinese Zheng, an instrument that none of the subjects could play. Under these circumstances, we may infer that the integration of visual and motor areas is not involved in composition. In a previous study, the visual and motor areas were activated during improvisation, probably due to the action of playing piano or the memory of playing piano while looking at the stave.

An important reason for concentrating on composing is that it is a process of free generation of individual notes^4^. On one hand, this free generation engages only creative thinking; thus, we suppose that extensive practice over a long period of time leads composers to develop a different distribution of brain networks, including generating music notes without the involvement of the visual and motor cortex. Music may have an impact on composers’ brain plasticity^26^,^27^,^28^. On the other hand, larger volumes of selected networks, along with a reduction in connectivity during the performance of the specified task, suggested that the recruitment of neurons for performing cognitive tasks might reduce hemodynamic coupling between brain regions^33^. It was found that auditory and motor networks are strongly linked in the musician’s brain, and even when the task involves only auditory or motor processing, co-activation of the areas can be observed^34^. It has also been found that auditory and visual cortices have the same strong relation when conducting musical imagery because auditory and visual imagery seem to obey similar basic neural principles^35^. In our study, we found that the functional connectivity of the visual and motor areas decreased. Perhaps in the real music creation task, the neurons of the visual and motor areas are recruited to connect with auditory cortex and to establish other functional connectivity with areas such as the ACC and the right angular gyrus.

### Why does the functional connectivity between the ACC and the DMN became stronger during composition?

We chose the ACC as the seed to calculate FC. As a result, we found that it had increased connectivity with the DMN. The ACC is known to be related to affect and motivation. It might also be related to other brain areas and could modulate cognitive, motor, endocrine and visceral responses^36^. However, some scientists have found involvement of the DMN in managing context-specific integration of information across large-scale brain systems^37^ and have shown a key effect on the integration of externally and internally directed control^38^. Thus, we speculate that the composing process may include not only the free generation of individual notes but also the combination of musical structures. Therefore, our results also support that the idea that DMN activity is related to only goal-irrelevant processes should be revised.

At this time, we also suggest that composing may include the combination of musical structures accompanying emotion in real time. The ACC plays an important role in the field of cognitive neuroscience. Lesions of the ACC produce a cluster of symptoms, including emotional instability^39^,^40^. Furthermore, one might assume that music creation requires activation of the prefrontal cortex as well as the ACC, the regions of the brain most closely associated with planning and higher cognitive function in humans^41^. During music composition, composers need to plan the combination of musical notes and emotion; composition is also an emotional task.

### Why was the effect of the lingual gyrus hub weakened during composition?

From Fig. 4, we can see that the effect of the lingual gyrus hub was weakened during composition. As we know, the lingual gyrus is usually involved in processing words. In a previous study, the lingual gyrus was reported to function in global shape processing during reading^42^. It has been demonstrated that activation of the lingual gyrus may be the greatest for low-contrast words and may decrease as contrast increases. Because music processing overlaps with language processing, these composers likely prefer to compose music note by note, rendering the lingual gyrus less critical during composition.

Imagined composition is limited in complexity compared to the real composition process. To investigate the real process, a composing device must be designed to fit in the MRI, and the procedure of task trials is needed. In addition, behavioral data, such as the training time for composition, was estimated by subjects and was thus somewhat arbitrary. Relatively precise training times should also be collected because the brain function of music creation may change with respect to training time.

## Conclusion

In summary, through our experiments, we found different brain functional connectivity in primary networks between the imagined composition state and the resting state among composers. The integration of visual and motor areas may not be involved in the real musical creation, and the neurons of these areas are recruited to enhance the functional connectivity between the ACC and the DMN for the purpose of planning the combination of musical notes and emotion. Nevertheless, further work should concentrate on the real composition task and should be a longitudinal study, so that we can delve deeper into the neural mechanism underlying composers’ brain plasticity and explore the genesis of creative behaviors.

## Additional Information

**How to cite this article**: Lu, J. *et al.* The Brain Functional State of Music Creation: an fMRI Study of Composers. *Sci. Rep.* **5**, 12277; doi: 10.1038/srep12277 (2015).

## Figures and Tables

**Table 1:** Changes between the resting state and the composition state in short- and long-range FCD regions (*p* < 0.001, cluster threshold *k* > 600 mm^3^).

	**Anatomical location (AAL template)**	**Voxels**	**MNI coordinate [X Y Z]**	**Peak t-score**
Short-range FCD	Occipital_Inf_R	122	[41 −81 −12]	−4.81
	Occipital_Inf_L1	45	[−21 −93 −7]	−5.33
	Occipital_Inf_L2	50	[−43 −74 −11]	−4.97
	Lingual_L	49	[−12 −64 −2]	−4.72
	Postcentral_L	234	[−51 −9 35]	−4.61
	Postcentral_R	102	[50 −9 36]	−4.12
Long-range FCD	Cingulum_Ant_L	45	[−4 39 3]	4.21
	Cingulum_Ant_R	35	[3 38 0]	4.65
	Postcentral_R	66	[58 −1 22]	−4.15
	Postcentral_L	166	[−60 −5 16]	−5.66
